# Composite hemangioendothelioma in the cervical spine with kaposiform hemangioendothelioma features in an elderly patient: a case report

**DOI:** 10.1186/s12877-022-03677-1

**Published:** 2022-12-09

**Authors:** Shunsuke Nakamura, Masashi Uehara, Shota Kobayashi, Hiromasa Hasegawa, Atsushi Tanaka, Jun Takahashi

**Affiliations:** 1 Department of Orthopaedic Surgery, Shinshu University School of Medicine, 3-1-1 Asahi, Matsumoto, Nagano, 390-8621 Japan; 2 Department of Laboratory Medicine, Shinshu University School of Medicine, 3-1-1 Asahi, Matsumoto, Nagano, 390-8621 Japan; 3 Hard Tissue Pathology Unit, Graduate School of Oral Medicine, Matsumoto Dental University, 1780 Gobara, Hirooka, Shiojiri, Nagano, 399-0781 Japan

**Keywords:** Composite hemangioendothelioma, Cervical spine, Elderly patient, Myelopathy, Surgical procedure, Radiotherapy

## Abstract

**Background:**

Composite hemangioendothelioma (CHE) is an intermediate group of tumors with features between hemangioma and angiosarcoma both histologically and biologically. CHE is predominant in young and middle-aged adults, but very infrequently affects the spine. We describe the case of primary CHE in the cervical spine exhibiting kaposiform hemangioendothelioma (KHE)-like components that was associated with cervical myelopathy with vertebral body destruction in an elderly woman. We retrospectively reviewed the case of a primary cervical spinal tumor, diagnosed as CHE with KHE-like components in pathological findings, associated with cervical myelopathy and cervical vertebral body destruction.

**Case presentation:**

An 80-year-old woman presented with progressive cervical myelopathy caused by a cervical spine tumor. Preoperative cervical MRI revealed a neoplastic lesion invading the cervical spine that strongly compressed the spinal cord, causing right upper-limb paralysis. We performed partial tumor resection along with posterior decompression and fixation. Postoperatively, pathological findings showed that the tumor was CHE with KHE-like features. Following radiotherapy, no recurrences have been observed in 21 months.

**Conclusions:**

This is the first report of CHE with features of KHE in the spine of an elderly patient. Posterior decompression and fusion of the cervical spine and subsequent radiotherapy resulted in a good outcome.

## Background

Composite hemangioendotherioma (CHE) is a rare intermediate endothelial tumor first described by Nayler et al. [1]. In the 2020 World Health Organization classification of Tumors of Soft Tissue and Bone, CHE was defined as a locally aggressive, rarely metastasizing vascular neoplasm containing a mixture of histologically distinct benign and malignant components [2]. The most common site of CHE is the soft tissues of the extremities or trunk, with spinal cases rarely reported [3–5]. The present patient is the oldest diagnosed case of CHE occurring in the spine.

## Case presentation

An 80-year-old woman presented to our hospital with the chief complaints of difficulty in raising her right upper limb, progressive dyskinesia, and gait disorder. She had been experiencing neck pain for approximately 2 years and numbness in her extremities for roughly a year before her visit, both of which had progressed remarkably prior to presentation. She could manage to walk unassisted for less than 100 m.

**Fig. 1:**
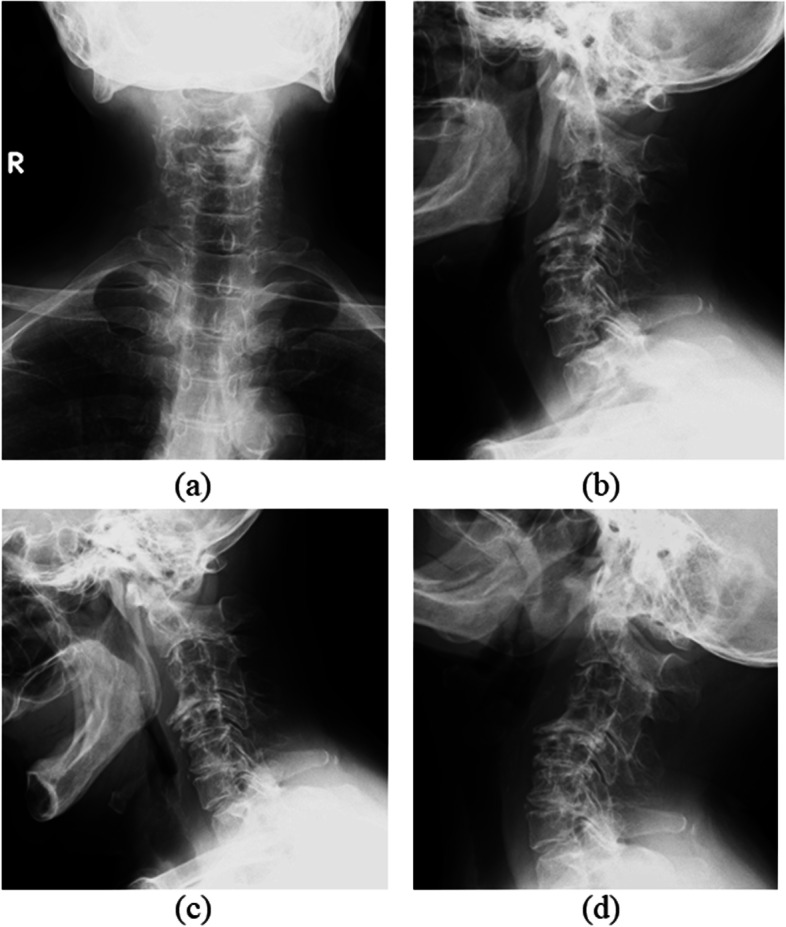
Preoperative radiographs. Radiographs showed osteolytic lesions in C3–7

**Fig. 2:**
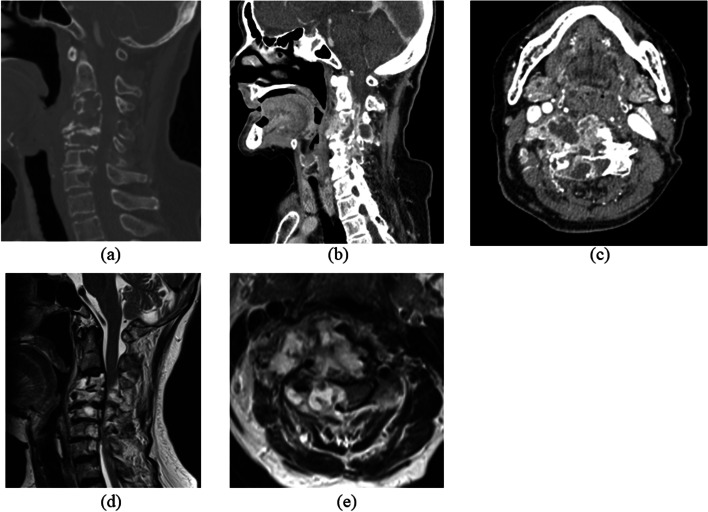
Preoperative CT and MRI. **a-c** Cervical contrast CT revealed osteolytic lesions in C3–7. The masses extended into the spinal canal from within the vertebrae, compressing the cervical spinal cord from the right side. **d**, **e** MRI T2-weighted imaging showed a mass with the same signal as bone from within the C3–7 vertebrae to outside the vertebral bodies. The cervical spinal cord at the same levels was compressed from the right side

**Fig. 3:**
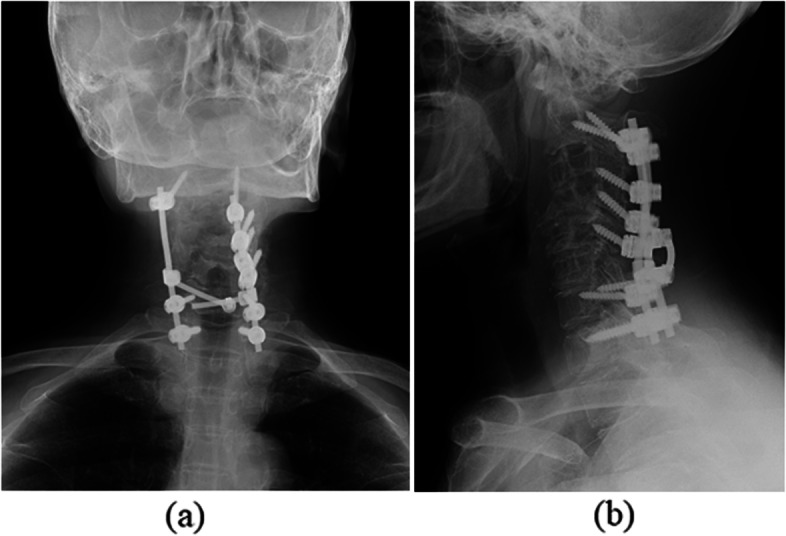
Postoperative radiographs. We performed posterior decompression and fixation from C2 to T1 and partial tumor resection to improve the patient’s cervical myelopathy symptoms

Regarding limb strength, muscle weakness (manual muscle testing [MMT] score: 3) was observed in the entire right upper limb. Her strength in the remaining limbs was normal. Hypesthesia was noted throughout the upper and lower limbs. Her deep tendon reflexes were increased bilaterally in all limbs, with positive Hoffman’s and Wartenberg’s signs. Her preoperative Japanese Orthopaedic Association (JOA) score was 8.5 points. Radiographs and cervical contrast CT revealed osteolytic lesions in C3–7. A mass was seen to extend into the spinal canal from within the vertebrae, compressing the cervical spinal cord from the right side. The mass was stained in the early contrast phase and showed an internal low-density area (Figs. 1 and 2a-c). MRI detected a mass with the same signal profile as bone from within the C3–7 vertebrae to outside of the vertebral bodies, and the cervical spinal cord was compressed from the right side at the same levels (Fig. 2d, e). Blood tests disclosed no thrombocytopenia, anemia, or coagulation abnormalities. Her inflammatory response was normal. Blood cultures and various tumor markers were negative. A percutaneous vertebral biopsy showed culture negativity and no neoplastic lesions. We ultimately performed posterior decompression and fixation of C2-T1 with tumor reduction to improve her cervical myelopathy symptoms (Fig. 3).

**Fig. 4:**
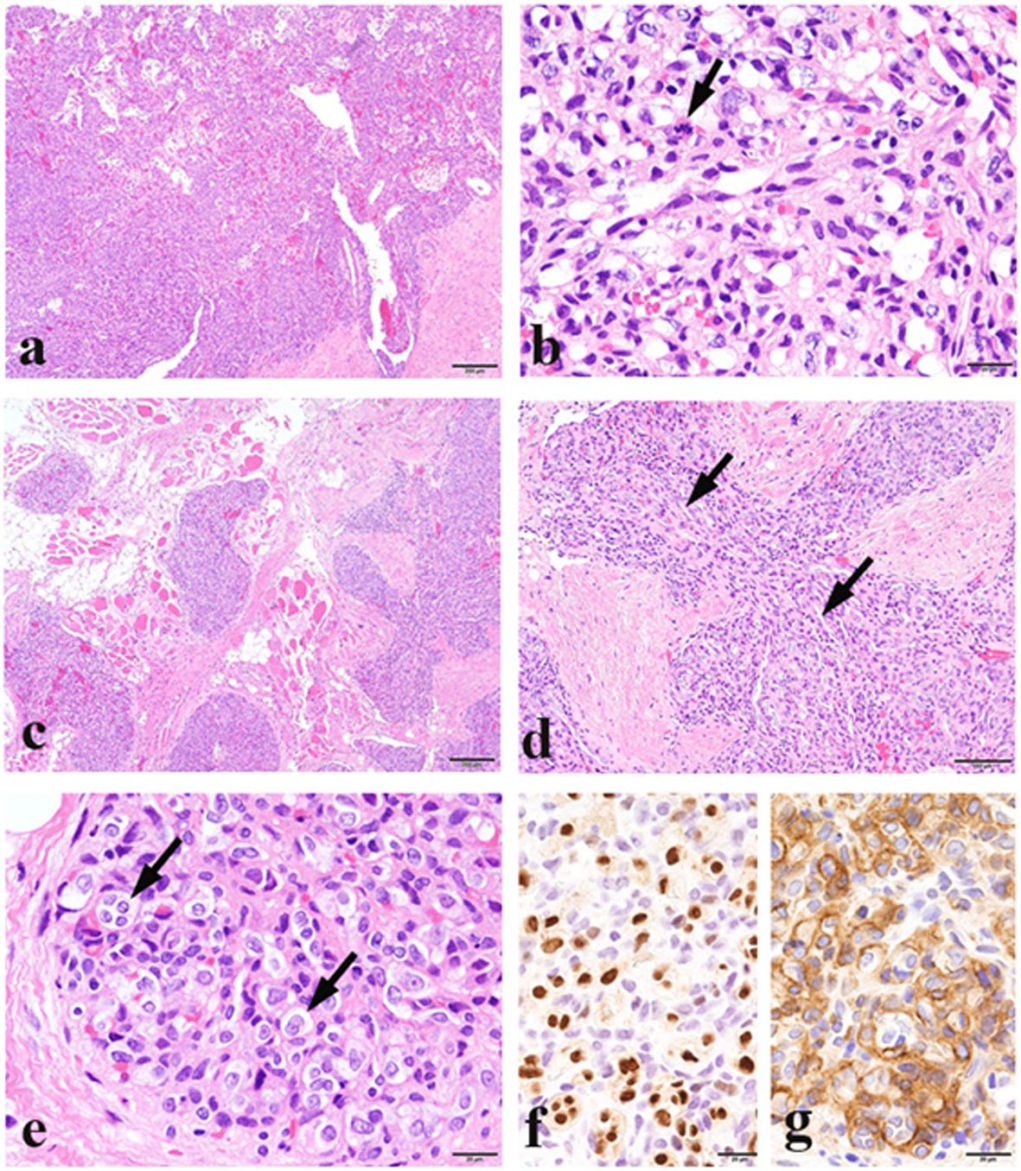
Histopathological findings. **a** Tumor showing solid and cavernous patterns (scale bar: 200 μm). **b** Atypical vasoforming cells displaying mitosis (arrow) in a solid area (scale bar: 20 μm). **c** Irregular nodules infiltrating the neighboring soft tissue (scale bar: 200 μm). **d** Slit-like structures (arrows) in the nodule containing spindle cells (scale bar: 100 μm). **e** Epithelioid endothelial cells (arrows) in some nodules (scale bar: 20 μm). **f** ERG-positive epithelioid cells (scale bar: 20 μm). **g** Myo1B-positive pericytes around epithelioid endothelial cells (scale bar: 20 μm)

In histopathological analysis, the resected specimen exhibited various and complex proliferating patterns of vasoforming cells in low-magnification images, with most areas showing a combination of solid and vasodilated proliferating patterns (Fig. 4a). In solid areas, numerous vascular cavities had been formed by atypical cells, including epithelioid cells with infrequent mitosis (Fig. 4b). In the periphery, irregular nodules had infiltrated into the adjacent muscular and fatty-fibrous tissue (Fig. 4c). The nodules had a slit-like structure with spindle cells (Fig. 4d). Several nodules contained epithelioid cell proliferation (Fig. 4e) that clearly showed ERG (Fig. 4f), CD31, and CD34, but not D2–40, immunopositivity, which were surrounded by cells positive for the pericytic markers myo1B (Fig. 4g), CD146, and SMA. The proliferating cells were negative for markers of epithelioid hemangioendothelioma, including CAMTA1, TFE3, and FOSB (data not shown). The Ki-67 labelling index was calculated as approximately 16.1% (109/678) in areas with solid components and dilated capillaries and 10.6% (83/783) in areas containing slit-like blood vessels in the solid components. The above histopathological features were compatible with a combination of low-grade angiosarcoma and kaposiform hemangioendothelioma (KHE)-like tumor. Considering the morphological and immunohistochemical findings, the patient was diagnosed as having CHE consisting of low-grade angiosarcoma-like and KHE-like components.

**Fig. 5:**
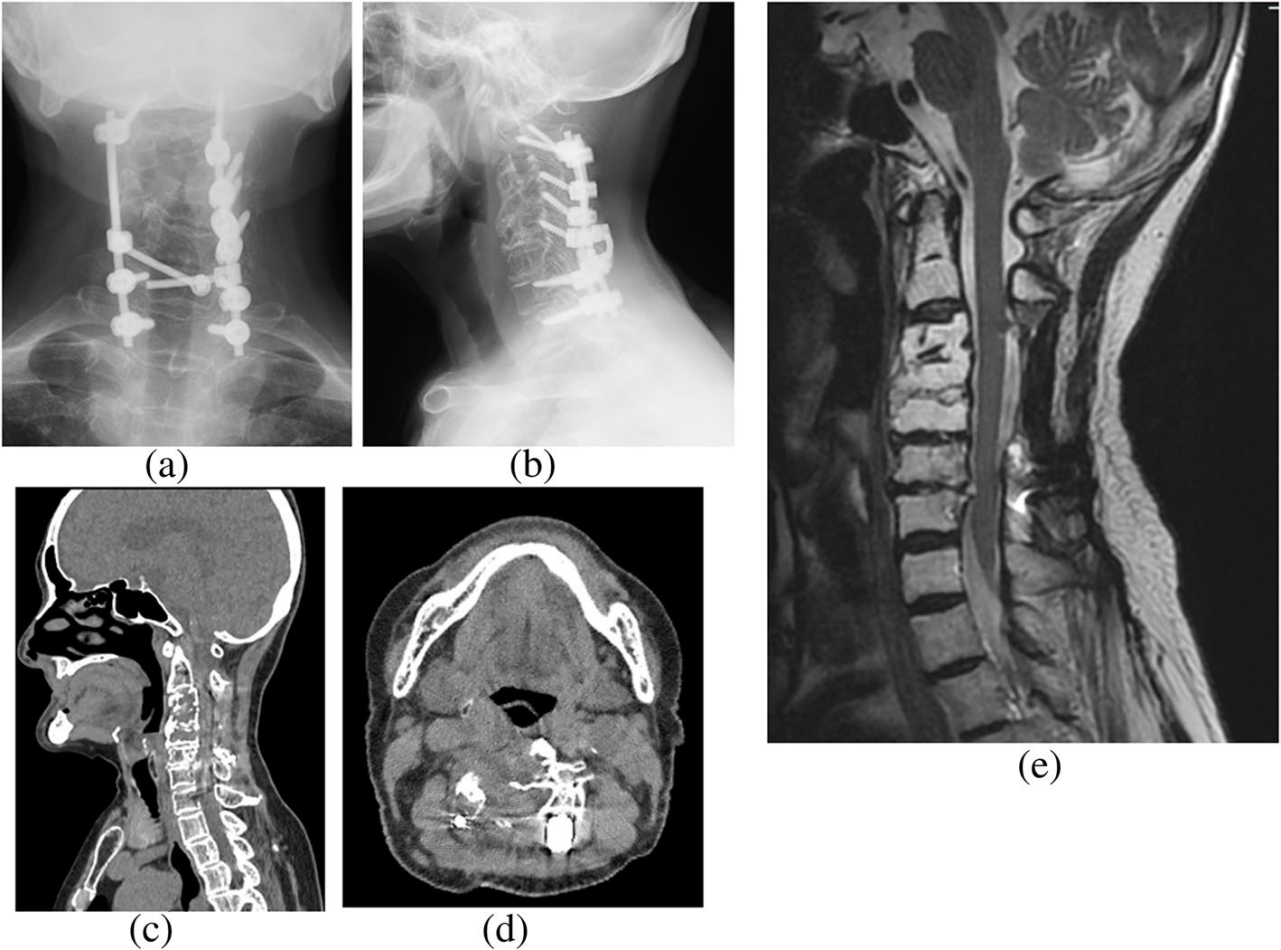
Radiographs, cervical CT, and MRI T2-weighted images at 18 months after surgery. There were no signs of recurrence and no significant changes in the osteolytic lesions on CT

Postoperatively, we commenced radiation therapy (50 Gy/25 Fr). At 21 months after surgery, her right upper extremity muscle strength had improved (MMT score: 5), she had no motor or sensory deficits in her extremities, and was able to walk steadily. Her JOA score improved to 17 points, with no signs recurrence in imaging findings (Fig. 5).

## Discussion and conclusion

To the best of our knowledge, this case is the first of spondylogenic CHE in an elderly woman. Although CHE commonly occurs in the skin and soft tissues of the extremities and trunk, it has also been reported in the periaortic region, pulmonary vein, liver, spleen, pancreas, lung, and right atrium [4, 6–8]. CHE generally afflicts young to middle-aged adults but can occur at any age [4, 7, 9]. The diagnosis of the present case was difficult since reports of CHE in the spine and bones are very rare. However, the combination of several pathological features led to the identification and successful treatment of CHE.

The diagnosis of CHE is challenging. Most cases present with various histological features, such as hemangioma, retiform HE, epithelioid HE, spindle HE, and low-grade angiosarcoma [1]. Shin et al. reported that the mean Ki-67 index was 4.6% for all hemangiomas and 38.1% for all angiosarcomas, with 15.0–17.5% as the cutoff value to distinguish between hemangioma and low-grade angiosarcoma [10]. In our patient’s specimen, most areas showed a combination of solid and vasodilated proliferating patterns with a variety of vascular cavities formed by atypical cells, including epithelioid cells with infrequent mitosis and a relatively low Ki-67 index (16.1%), as evidence of angiosarcoma. These findings supported our diagnosis of the partial presence of low-grade angiosarcoma-like features. Although the spindle cell component in CHE is not rare [1, 2], the patient had solid and irregular nodules infiltrating into the adjacent muscular and fatty-fibrous tissue in the periphery. Some nodules included epithelioid endothelial cell proliferation surrounded by myo1B-, CD146-, and SMA-positive pericytes, which were considered as an epithelioid hemangiomatous component. As Lyons documented that KHE comprises scattered glomeruloid nodules containing epithelioid endothelium and αSMA-positive pericytes in addition to spindle cell proliferation [11], our case also partially resembled KHE. Considering the complex morphology of proliferating cells, our patient was diagnosed as having CHE with KHE-like features.

A review of the literature identified a total of 52 patients with CHE (mean age: 42.5 years) reported in the English language, including a 48-year-old woman with CHE in the spine [12]. Most patients underwent surgical treatment. Radiotherapy, chemotherapy, electron beam irradiation, interferon-α 2b, and thalidomide have also been described as effective for CHE with or without resection, although are less common than surgery [12–17]. Since CHE in the spine or bones is rare, there remains no consensus on its treatment [13, 14]. Dong et al. described a case of a 56-year-old man with CHE in the manubrium sterni, in which lesion resection was performed [3]. Gok et al. reported a case of CHE in paravertebral muscle in a 54-year-old man also treated by excision only [18]. In contrast, Mani et al. described a case of CHE in the calcaneus, distal femur, proximal tibia, and patella of the left leg, as well as a case of CHE in the left fibula and foot, which were treated with radical resection by above-knee and below-knee amputation, respectively [5]. Cheuk et al. encountered a case of CHE in paravertebral soft tissue that was prone to recurrence after resection [19].

Mahmoudizad et al. reported that radiotherapy for CHE on the scalp resulted in tumor shrinkage [13]. Sakamoto et al. performed surgical resection of CHE in the foot and radiotherapy alone for CHE in the lower extremity arteries. The lower-extremity CHE shrunk in size and had not recurred at 2.5 years [14]. In the present case, the tumor could not be completely resected, which necessitated postoperative radiation therapy. No evidence of tumor re-growth was seen at the most recent follow-up. Additional cases are needed to confirm the efficacy of radiation therapy for CHE.

Considering that CHE is an intermediate-malignant tumor, there is a need for postoperative adjuvant treatment. In our case, the patient had cervical myelopathy symptoms due to severe cervical cord compression by the tumor and was treated surgically and with postoperative radiation to prevent recurrence. No sequalae have been observed for 21 months since surgery. However, longer observation for tumor recurrence and radiation myelopathy is necessary.

This is the first report of CHE with KHE components arising in the spine of an elderly patient. Posterior decompression fixation of the cervical spine and tumor enucleation for progressive cervical myelopathy followed by radiotherapy resulted in good symptomatic improvement without recurrence.

## Abbreviations

CHE: Composite hemangioendotherioma
MMT: $manual muscle testing
JOA: Japanese Orthopaedic Association
KHE: Kaposiform hemangioendothelioma
HE: Hemangioendothelioma
